# In Vivo Efficacy Testing of Peptide Receptor Radionuclide Therapy Radiosensitization Using Olaparib

**DOI:** 10.3390/cancers15030915

**Published:** 2023-02-01

**Authors:** Danny Feijtel, Thom G. A. Reuvers, Christine van Tuyll-van Serooskerken, Corrina M. A. de Ridder, Debra C. Stuurman, Erik de Blois, Nicole S. Verkaik, Peter de Bruijn, Stijn L. W. Koolen, Marion de Jong, Julie Nonnekens

**Affiliations:** 1 Department of Radiology and Nuclear Medicine, Erasmus MC Cancer Institute, Erasmus University Medical Center, 3015 GD Rotterdam, The Netherlands; 2 Department of Molecular Genetics, Erasmus MC Cancer Institute, Erasmus University Medical Center, 3015 GD Rotterdam, The Netherlands; 3 Department of Urology, Erasmus University Medical Center, 3015 GD Rotterdam, The Netherlands; 4 Department of Medical Oncology, Erasmus MC Cancer Institute, Erasmus University Medical Center, 3015 GD Rotterdam, The Netherlands; 5 Department of Hospital Pharmacy, Erasmus University Medical Center, 3015 GD Rotterdam, The Netherlands

**Keywords:** Poly(ADP-ribose)-polymerase inhibition, peptide receptor radionuclide therapy, radiosensitization, olaparib

## Abstract

**Simple Summary:**

Neuroendocrine tumor (NET) patients often suffer from metastases, thereby eliminating surgery as a curative treatment option. A possible treatment strategy for these patients is peptide receptor radionuclide therapy (PRRT). PRRT is composed of a radiolabeled peptide that can bind to the NET-cells via a specific receptor. After intravenous injection of the radiolabeled peptide and binding to the NET cells, the radionuclide induces DNA damage upon radioactive decay, leading to cell death. However, the majority of patients will not be cured with the current regimen. Therefore, there is an urgent need for therapy improvement. Previously, it was shown, in cell models, that the combination treatment of PRRT with a poly(ADP-ribose)-polymerase (PARP) inhibitor, which inhibits DNA damage repair, can be effective. As the next step towards patients, we have tested this combination treatment in animal models and showed that, in mice, the combination of PRRT with PARP inhibitors is more effective than PRRT alone, however not in all the tested models. This discrepancy is of importance for the translation of this type of therapy towards the clinic.

**Abstract:**

Peptide receptor radionuclide therapy (PRRT), a form of internal targeted radiation treatment using [^177^Lu]Lu [DOTA^0^-Tyr^3^]octreotate, is used to treat patients with metastasized neuroendocrine tumors (NETs). Even though PRRT is now the second line of treatment for patients with metastasized NETs, the majority of patients will not be cured by the treatment. PRRT functions by inducing DNA damage upon radioactive decay and inhibition of DNA damage repair proteins could therefore be used as a strategy to potentiate PRRT. Previous work has shown promising results on the combination of PRRT with the PARP inhibitor olaparib in cell lines and mice and we have been taken the next step for further in vivo validation using two different xenografted mouse models. We observed that this combination therapy resulted in increased therapeutic efficacy only in one model and not the other. Overall, our findings indicate a tumor-type dependent anti-tumor response to the combination of PRRT and olaparib. These data emphasize the unmet need for the molecular stratification of tumors to predetermine the potential clinical value of combining PARP inhibition with PRRT.

## 1. Introduction

Peptide receptor radionuclide therapy (PRRT) is a form of internal radiation to treat patients with metastasized neuroendocrine tumors (NETs). PRRT relies on the binding of the peptide octreotate with high affinity to the somatostatin receptor subtype 2 (SSTR_2_), which is highly overexpressed on NET cells. PRRT consists of the somatostatin analogue octreotate, coupled to a DOTA-chelator, labeled with the radiometal lutetium-177. After injection in the circulation, NET lesions will be specifically targeted and DNA damage will be induced during radioactive decay leading to NET cell death [1]. This compound, [^177^Lu]Lu[DOTA^0^-Tyr^3^]octreotate (^177^Lu-DOTA-TATE) is EMA and FDA approved for treatment for metastatic gastroenteropancreatic NETs (GEP-NETs) and holds great promise in terms of the improvement of progression free survival and quality of life [2]. However, the vast majority of ^177^Lu-DOTA-TATE treated patients will not be cured at this stage of the disease.

As PRRT is currently non-curative in metastatic GEP-NETs, this has prompted several investigations into improvement strategies. These strategies include altering the administered doses or treatment schedules [3], using other radiometals with different radiation properties [4], or combining PRRT with other chemotherapeutic drugs such as carboplatin or etoposide (reviewed in [5]). Previously, we have shown the synergistically enhanced anti-cancer effect of using PRRT combined with the DNA damage repair inhibiting drug olaparib [6].

Olaparib restricts the targeted cells from repairing radiation-induced DNA single strand breaks (SSBs), which will subsequently be converted to the more cytotoxic DNA double strand breaks (DSBs) during replication. The proof of concept for the synergistic anti-cancer effect of this combinatorial strategy has previously been published in different preclinical models [6,7,8]. Investigative efforts into the combination of PARP inhibitors and PRRT are also extending towards other malignancies, such as prostate cancer, and show increasing promise [9,10]. However, the exact mechanism of action remains elusive as current knowledge is mainly restricted to therapeutic response in vitro or limited in vivo studies. For this reason, we investigated the synergistic tumor killing potential combining PRRT and olaparib in two SSTR_2_-expressing cancer cell models in vivo. Interestingly, we found that the proof-of-concept of this combinatory strategy is not successful in both models. This emphasizes the need for further investigation into molecular factors that might impede the efficacy of olaparib-PRRT combination therapies.

## 2. Materials and Methods

### 2.1. Cell Culture Conditions

All cell culture media were supplemented with penicillin (50 units/mL, Sigma-Aldrich, Zwijndrecht, The Netherlands), streptomycin (50 µg/mL, Sigma-Aldrich) and 10% fetal calf serum (Biowest, Nuaillé, France). NCI-H69 cells (ATCC) were cultured in Rosewell Park Medium Institute 1638 medium (Sigma-Aldrich, Zwijndrecht, The Netherlands). CA20948 cells [11] were cultured in Dulbecco’s Modified Eagle’s Medium (Gibco, Bleiswijk, The Netherlands). All cells were cultured at 37 °C and 5% CO_2_.

### 2.2. Animal Experimental Conditions and Tissue Collection

All performed animal experiments were approved by Erasmus MC’ Animal Welfare Committee and have been conducted according to European Guidelines. The mouse survival studies were conducted using xenografted solid-tumor models in BALB-c/nude mice. For this 5 × 10^6^ NCI-H69 cells were inoculated subcutaneously on the left flank in 200 µL Hanks Balanced Saline Solution (HBSS, Gibco, Bleiswijk, The Netherlands) containing 33.3% matrigel (Corning, Amsterdam, The Netherlands). In other mice, 5 × 10^6^ CA20948 cells were inoculated subcutaneously on the left flank using 200 µL HBSS. Tumor volumes were measured by calipers. For all animal experiments, mice were injected intravenously with 30 MBq/0.5 µg ^177^Lu-DOTA-TATE (diluted in PBS containing 0.1% bovine serum albumin (BSA, Sigma-Aldrich)), which was labeled as previously described with a purity of >95% [12]. Animals were treated intraperitoneally with either olaparib (Selleckchem, Planegg, Germany) (50 mg/kg dissolved in 4% DMSO (Sigma-Aldrich), 30% PEG300 (Sigma-Aldrich), 66% dH_2_O) or its vehicle control for 14 consecutive days, starting two days prior to PRRT injection. Mice for downstream molecular analyses (*n* = 4 per group) were sacrificed by cervical dislocation on day 2 or 4 post injection (p.i.) of PRRT, and animals that were used for survival analyses were sacrificed when tumors reached size of 2000 mm^3^, for maximum 90 days p.i. or when humane endpoints were reached at the end of the experiment (CA20948: vehicle; *n* = 8, olaparib; *n* = 12, PRRT; *n* = 12, PRRT + olaparib; *n* = 13) (NCI-H69: all groups; *n* = 11). For survival experiments data was collected and pooled with previously generated data [13] (previously collected NCI-H69 data: vehicle *n* = 9, PRRT *n* = 8; previously collected CA20948 data: vehicle *n* = 6, PRRT *n* = 9). Blood was collected by intracardiac puncture during sacrifice and was centrifuged for 10 min at 4024× *g* for 10 min at 4 °C in heparin-lithium vials (MiniCollect), after which obtained plasma was snap frozen in liquid N_2_ and stored at −80 °C. Excised tumor tissues were either fixed and weighed in formalin after which gamma-counts were measured in a gamma-counter (PerkinElmer, Hoogvliet, The Netherlands) and were processed further or snap frozen in liquid N_2_ and stored at −80 °C for downstream analyses. The analyzed time to progression (TTP) depicts the length of time (days) of the median growth of a tumor from the start of treatment until the endpoint of a subject in the experiment.

### 2.3. Olaparib Measurements

Olaparib concentrations in plasma and tissues were analyzed using a validated Ultra Performance Liquid Chromatographic method coupled to tandem mass spectrometry (UPLC-MS/MS). Briefly, olaparib was extracted from 25 μL aliquots of plasma after the addition of 100 μL Internal Standard Working Solution (100 ng/mL dasatinib-d8 in acetonitrile). After vigorously mixing for 5 s and centrifugation for 10 min at 18,000× *g*, an aliquot of 50 µL of the clear supernatant was transferred into a 700-µL 96-well plate and 100 µL of water/formic acid/ammonium formate (100:0.1:0.02, *v*/*v*/*v*) was added. After mixing, 2 μL was injected into the UPLC-MS/MS system. Calibration curves were constructed in human plasma and were linear over the range of 50 to 5000 ng/mL with the lower limit of quantitation validated at 50.0 ng / mL. Tissues were homogenized in 200 µL of blank human plasma with a tissue-lyser (Qiagen, Germany) and a stainless-steel bead (5 mm) for 90 s at 50 Hz. Homogenized tissue samples were further processed as plasma samples, as described above.

### 2.4. Tissue Processing and Immunofluorescent Stainings

Tumor tissues that were to be used for immunofluorescent staining (IF) were fixed in formalin for one day at room temperature (RT). Tissues were then processed, dehydrated and embedded in paraffin using a tissue processor (RTPH-360, General Data Healthcare). IF was performed on 4 µm cut paraffin embedded tissues. Tissue sections were first deparaffinized and rehydrated. Antigen retrieval was then performed by boiling sections for 20 min in pH 9 antigen retrieval buffer (DAKO, S2368). Then, sections were permeabilized using 0.5 % triton X-100 (Sigma-Aldrich) in PBS (PBS-T) for 10 min at RT and then incubated in blocking buffer (3% BSA in PBS-T) for 30 min at RT. Sections were then incubated in blocking buffer containing the primary antibody (phosphorylated histone 2A (γH2AX) (Millipore, Amsterdam, The Netherlands, JBW301; 1:250)) for 90 min at RT. After washing, the sections were incubated in blocking buffer containing the secondary antibody for 45 min at RT. The sections were mounted using Vectashield containing DAPI (Vector Labs, Newark, CA, USA, H-1200).

### 2.5. Statistical Analyses

Statistical analyses were performed in GraphPad Prism version 9.3.1. For determining significance Student *t*-tests or one-way ANOVA followed by Browne-Forsythe and Welch posttest when samples were compared to a single control. When samples were compared to each other, a one-way ANOVA was performed followed by Tukey’s or Bonferroni’s posttest. All tests with *p* values < 0.05 were deemed significant.

## 3. Results

### 3.1. Olaparib Enhanced the Induction of DNA Damage during PRRT in Mice Bearing CA20948 Tumors

**Figure 1:**
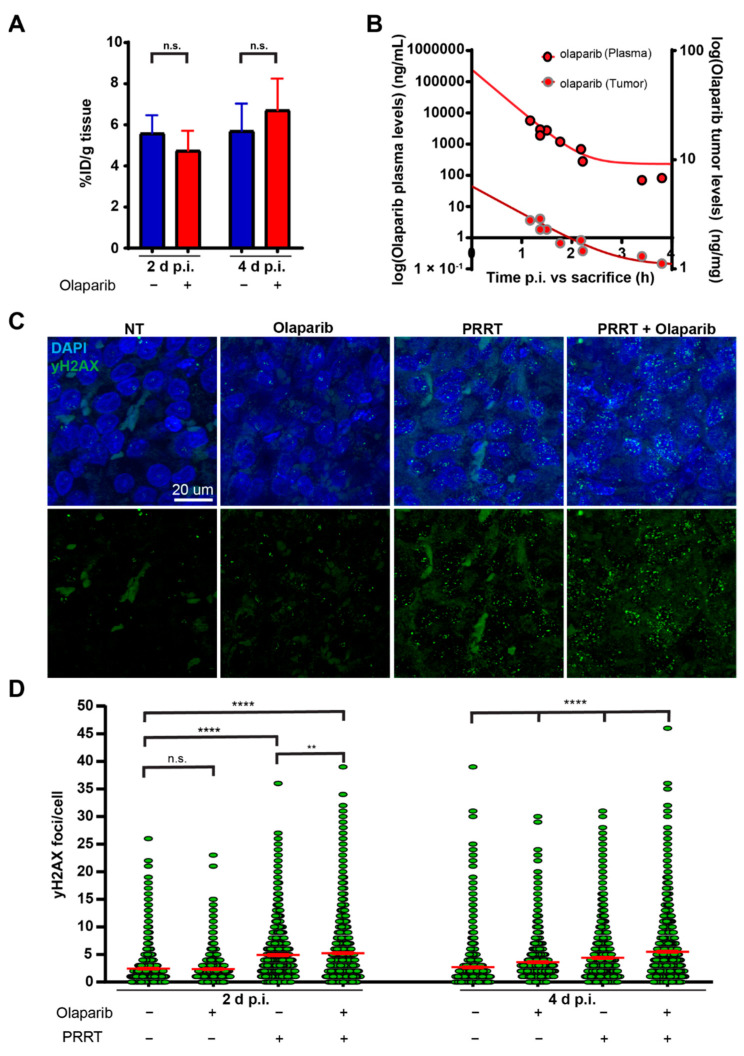
Olaparib in combination with PRRT increases DNA damage in CA20948 tumors. (**A**) Measured radioactivity in CA20948 tumors on two days and four days p.i. in the percentage of injected dose per gram (% ID/g). (**B**) Olaparib concentrations in ng/mL measured in plasma (red with black dots) and in ng/mg in CA20948 tumors (red with grey dots) from different mice on different time-points after injection. (**C**) Representative images of DNA damage marker γH2AX (green) on IF stained tumor sections on day 4 p.i. Nuclei are stained with DAPI (blue). (**D**) Quantification of the number of γH2AX foci per cell in CA20948 tumors two days and four days p.i. (*n* = 3). Error bars indicate the standard error of the mean. ****: *p* < 0.0001; **: *p* < 0.01; n.s.: not significant.

Previously, we have shown that CA20948 cells can be radiosensitized to PRRT using olaparib [6]. To investigate whether this can be recapitulated in vivo, we engrafted CA20948 cells on immunocompromised mice. These animals received daily injections with vehicle or olaparib starting two days before PRRT and were sacrificed two days or four days post PRRT injection. To ensure that the potential radiosensitizing effects of olaparib were not due to enhanced radioactive uptake, radioactive uptake in tumors from the PRRT-injected mice was measured using a gamma-counter after sacrifice and tumor excision. The percentage of injected dose per gram of lutetium-177 in tumors from PRRT mice did not differ significantly between olaparib and vehicle treated mice (Figure 1A). In addition, we examined the bioavailability of olaparib in both plasma and tumors upon animal sacrifice by HPLC. Here, a strong correlation was observed between the time of olaparib injection and the moment of olaparib measurement (Figure 1B). All animals that were treated with olaparib showed measurable olaparib levels in both their plasma and tumors (Figure 1B). No trace of olaparib could be detected in vehicle-treated animals.

In order to examine whether olaparib increases the number of DSBs during PRRT, we performed immunofluorescent stainings of phosphorylated histone 2A (γH2AX) on tumor sections (Figure 1C). γH2AX forms nuclear foci at the site of a DSB and are therefore a good marker to quantify the number of DSBs [14]. We observed that PRRT induces γH2AX foci formation, which remains significantly higher than the vehicle or olaparib controls on both two and four days post injection (Figure 1D). At both time points, we did find a small, but significant increase in the number of γH2AX foci in the tumors of combination treated mice compared to PRRT alone. On 4 days post injection, tumors from mice that had received the combination treatment retained significantly more DNA damage than PRRT alone. Olaparib controls showed a significant elevation of the number of γH2AX foci after four days post injection compared to the vehicle controls.

### 3.2. Combination of PRRT and Olaparib Synergistically Improves CA20948 Tumor Control

**Figure 2:**
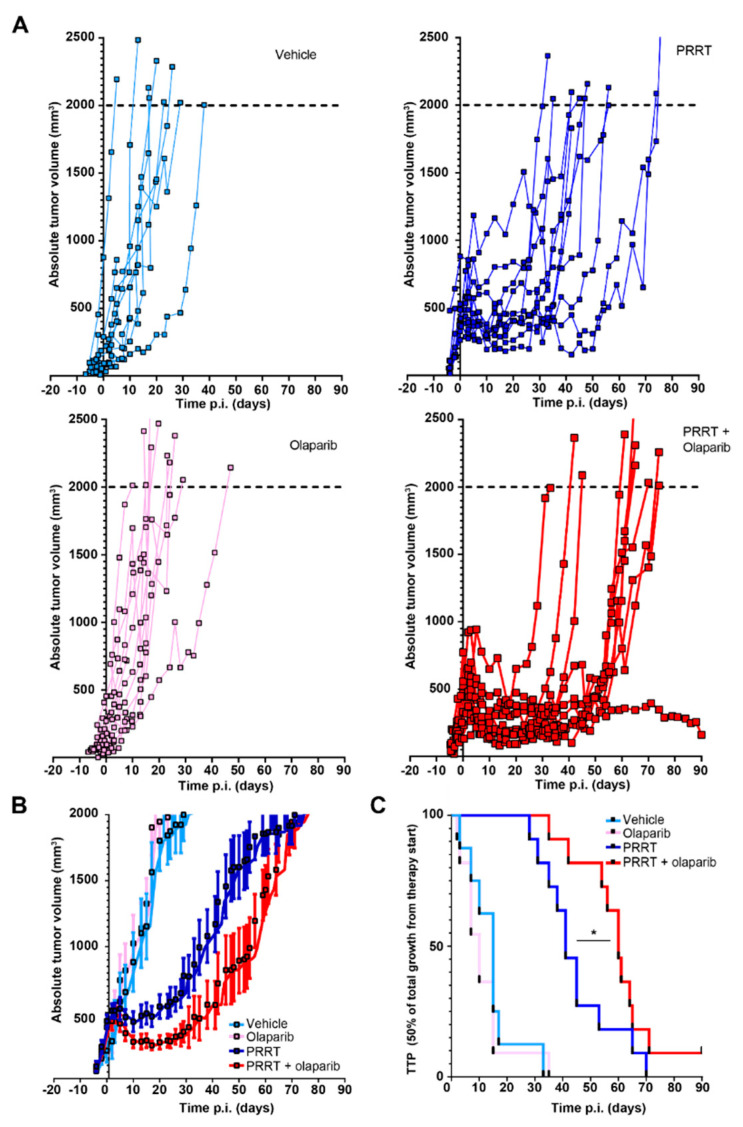
Combination of PRRT and olaparib synergistically enhances tumor control in vivo in mice bearing CA20948 tumors. (**A**) CA20948 tumor growth curve showing absolute tumor volumes of mice that were treated with vehicle, olaparib, PRRT or the combination of PRRT and olaparib. (**B**) Combination of all absolute tumor volumes per treatment group. Error bars indicate Standard error of the mean. (**C**) TTP shown by Kaplan-Meijer curves of CA20948 tumor bearing mice that were treated with vehicle, olaparib, PRRT or the combination of PRRT and olaparib. * *p* < 0.05.

As we observed that the combination of olaparib and PRRT increased the number of DSBs in CA20948 bearing animals, we set out to investigate whether this effect would elicit improved tumor control. Again, CA20948 cells were engrafted and the mice were treated with vehicle, olaparib, PRRT or the combination of PRRT and olaparib. No difference in response between the vehicle and olaparib groups was observed. PRRT did induce a delay in tumor growth compared to both control groups (Figure 2A,B). In the vehicle and olaparib control groups, the time to progression (TTP) median was observed to be 15 and 10 days, respectively (Figure 2C). In the PRRT group, the TTP median observed was 41 days.

Importantly, when the olaparib treatment was combined with PRRT, we did observe an increase in tumor control compared to PRRT alone, with even achieving complete tumor control in one animal, at least until the end of the experiment (90 days after PRRT injection) (Figure 2A,B). A significant improvement of PRRT and olaparib combination treatment was observed compared to PRRT alone as the tumor control and median TTP increased from 41 to 60 days (Figure 2C).

Acute toxicity of the treatments was assessed by monitoring the body weights of the mice. Here, we observed a temporary decline in body weight after PRRT injections. However, after approximately 10 days the mice started recovering and the majority gained weight again. No significant differences were observed in body weight decline and recovery between the PRRT alone and combination therapy group (Supplementary Figure S1A).

### 3.3. Combining PRRT with Olaparib Does Not Improve Tumor Control in NCI-H69 Tumors

To verify the findings of combining PRRT with olaparib in a second xenograft model, we used the SSTR_2_-expressing NCI-H69 cell line. Previously, we have documented dosimetric calculations and associated effects of PRRT on NCI-H69 tumors in vivo [13]. In order to test the potential radiosensitizing effect of olaparib, we applied the same treatment regimens as with CA20948 tumors.

**Figure 3:**
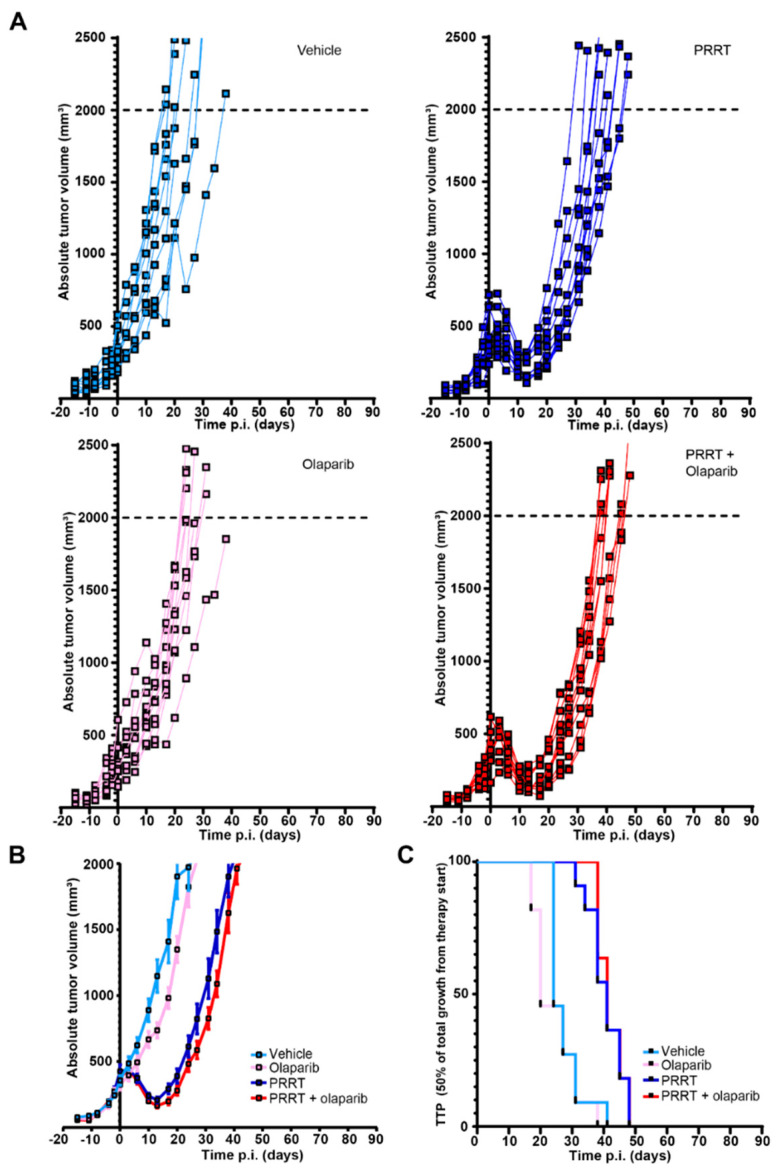
Combining PRRT and olaparib does not increase therapeutic efficacy compared to PRRT monotherapy in mice bearing NCI-H69 tumors. (**A**) NCI-H69 tumor growth curves showing absolute tumor volumes of mice that were treated with vehicle, olaparib, PRRT or the combination of PRRT and olaparib. (**B**) Combination of all absolute tumor volumes per treatment group. Error bars indicate the Standard error of the mean. (**C**) TTP shown by Kaplan-Meijer curves of NCI-H69 tumor bearing mice that were treated with vehicle, olaparib, PRRT or the combination of PRRT and olaparib.

In concordance with previous experiments [13], we observed a decline in tumor volumes approximately four until eleven days post injection of PRRT in the PRRT alone and combination treatment mice (Figure 3A,B). However, we observed no increase in tumor control in the combination treatment group compared to PRRT alone in these NCI-H69 tumor bearing mice. Olaparib alone did show a small, but non-significant effect on tumor control in the control and PRRT condition. In line with this, the TTP median of both the PRRT and the combination treatment groups was 41 days (Figure 3C), indicating that for mice bearing NCI-H69 tumors no beneficial effects were observed using the combination of PRRT and olaparib, in sharp contrast to the mice bearing CA20948 tumors. In addition, same as for the CA20948 tumor bearing mice, the combination of PRRT and olaparib also did not have any adverse effects in this cohort of mice (Supplemental Figure S1B).

## 4. Discussion

In this study, we investigated the anti-tumor effect of PARP inhibition as radiosensitizer of PRRT. We observed similar in vivo tumor responses to PRRT as we previously described [10] in both NCI-H69 and CA20948 tumors. We found that olaparib is able to sensitize CA20948 tumors, but not NCI-H69 tumors, to PRRT. This difference in therapeutic response and its possible underlying mechanisms of action might be an important prospect for the clinical situation and therefore warrants further investigation.

Currently, similar investigations into the combination of PARP inhibiting drugs and PRRT are performed in other malignancies, such as prostate cancer [9,10]. One study showed a synergistic increase in induced DSBs for the combination of PRRT and veliparib, but not for olaparib and talazoparib [9]. This shows the need for further molecular analysis into such discrepancies, especially as clinical trials using PARP inhibitors and radionuclide therapy are also being conducted (NCT03076203, NiraRad trial; NCT03874884, Lu-PARP trial).

The possible synergistic effect of PRRT and PARP inhibition on tumor killing has been shown by us and others in different models, both in vitro and in vivo [6,7,8], yet the mechanism of action is not fully understood and discrepancies between findings exist; such as when comparing our NCI-H69 and CA20948 tumor models. Various mechanisms could underlie these discrepancies. First, it is unclear whether potentiation of radiation treatment is possible if tumor cells are relatively radioresistant. Different (pre)clinical studies show that uptake of SSTR_2_-mediated radiopharmaceuticals did not always correlate with therapeutic efficacy [8,15,16]. In a recent investigation, xenografted mice bearing medulloblastoma tumor cells showed a complete response to PRRT in vivo. In the same study, another xenograft model using H1299-7 cells showed three times more [^68^Ga]Ga-DOTA-TATE uptake, however with worse tumor control and all subjects reaching their humane endpoints [8]. This discrepancy shows that different tumors can respond differently to a certain injected dose. Less radiosensitive tumors might, for example, have a relatively higher DNA repair activity, thereby rendering these tumors potentially less sensitive for DNA repair inhibiting drugs such as olaparib. Whether this is the case for the NCI-H69 tumors compared to CA20948 has to be determined in follow-up studies. However, since NCI-H69 cells did respond to PRRT it is not likely that radioresistance is a pivotal player in the success of combining PRRT and PARP inhibition in the current study. Additionally, it might be that other types of DNA damage repair are more prominent in certain tumors, compared to others, creating a bias for sensitivity to specific DNA repair inhibiting drugs [17,18,19,20].

Second, the proliferation rate of different tumor cells could impact radiosensitization, especially in the context of drugs that affect DNA integrity. It has already been shown that a slow-cycling fraction of human colorectal carcinoma cells exhibit a multifold resistance to oxaliplatin and 5-fluoro-uracil compared to the faster cycling fractions in the same culture [21]. This drug resistance implies a potential role for DNA replication speed in the sensitivity to DNA damage inducing drugs. Therefore, the question remains if potentiation of PRRT is possible at all with drugs that can affect DNA replication, in slow cycling cells. As the majority of patient NETs are slowly cycling tumors, this question is especially important for future clinical settings as the combination of PARPi and PRRT might be better suited for neuroendocrine carcinomas [22]. In light of this, NCI-H69 cells have a doubling time of 56 h [23], compared to CA20948 cells which double every 22 h [24]. This difference could potentially cause a difference in response to PARP inhibition. However, in both tumor models, no significant effect on tumor growth delay was observed for olaparib alone. However, both tumor models did respond to PRRT. Additionally, in another investigation, subpopulations of NCI-H69 cells with similar proliferation rates showed different chemosensitivities [25]. Here, a mesenchymal variant (NCI-H69V) showed higher chemoresistance compared to their more epithelial counterparts, whilst doubling times of both lines remained comparable. This indicates that the effects of proliferation speed on the effectiveness of chemotherapeutics might be secondary to underlying biological differences between tumor cells. However, the effect of proliferation speed on the therapeutic efficacy of combining PRRT with PARPi needs further investigation.

Not only can cellular context, such as cycling rate or radioresistance, hamper the potentiation of PRRT using PARP inhibitors, but there are also direct mechanisms of PARP inhibitor resistance which can have the same effect. For example, upregulation or basal expression levels of P-glycoprotein (P-gp) efflux pumps on the cell membranes can cause multidrug resistance including resistance to olaparib, rucaparib and paclitaxel [26]. In line with this, inhibition of P-gp efflux pumps can re-sensitize tumors to olaparib [27]. Moreover, a variant of olaparib that lacks affinity for P-gp efflux pumps, AZD2461, minimizes PARPi resistance, emphasizing the impact of multidrug resistance proteins [28]. Another mode of PARPi resistance might be the removal of trapped PARP-1 on DNA damage lesions, the most cytotoxic mode of action of olaparib [29]. During s-phase, trapped PARP-1 can interfere with the replication machinery and cause stalled replication forks to collapse. The removal of trapped PARP-1 has been described as a mode of PARPi resistance. In concordance, investigations have shown that the expression of helicases that are able to remove trapped PARP-1 from the DNA, can hamper the therapeutic efficacy of olaparib [30]. It might not be feasible to screen patient tumors for the expression of transporter proteins or expression of PARP-1 removing helicases just yet, but it does entail interesting avenues of research. This should also be investigated in NCI-H69 and CA20948 cells, as currently the presence of these PARP-1 resistance factors has, to our knowledge, not yet been investigated.

In conclusion, the combination of DNA damage repair modulating drugs with PRRT is a promising therapeutic avenue. However, factors that can mitigate efficacy and potentially lead to resistance need to be taken into account when performing combination studies, both in vitro and in vivo. Such insights might improve potential clinical studies where PRRT is combined with drugs such as olaparib.

## 5. Conclusions

We show the possibility of olaparib-PRRT combination treatment in the CA20948 xenografted mouse model, and show the lack thereof in the NCI-H69 xenografted mouse model. Further molecular stratification is warranted to anticipate therapeutic discrepancies in patients.

## Data Availability

The data that support the findings of this study are available from the corresponding author, J.N., upon reasonable request.

## Supplementary Materials

The following supporting information can be downloaded at: https://www.mdpi.com/article/10.3390/cancers15030915/s1, Figure S1: Toxicity assessment of different treatment groups from CA20948 and NCI-H69 tumor experiments.

## Abbreviations

^177^Lu-DOTA-TATE	[^177^Lu]Lu[DOTA^0^-Tyr^3^]octreotate
BSA	Bovine serum albumin
γH2AX	Phosphorylated histone 2A
DAPI	4’,6-diamidino-2-phenylindole
DSB	Double strand break
DMSO	Dimethyl sulfoxide
GEP-NETs	Gastroenteropancreatic-neuroendocrine tumors
HBSS	Hanks balanced salt solution
IF	Immunofluorescent
NETs	Neuroendocrine tumors
PARP	Poly(ADP-ribose)-polymerase
PARPi	Poly(ADP-ribose)-polymerase inhibitor
PBS	Phosphate buffered saline
PBST	Phosphate buffered saline containing Triton X-100
PEG300	Polyethylene glycol 300
P-gp	P-glycoprotein efflux pump
P.i.	Post-injection
PRRT	Peptide receptor radionuclide therapy
SSB	Single strand break
SSTR_2_	Somatostatin receptor subtype 2
TTP	Time to progression
UPLC-MS/MS	Ultra performance liquid chromatography-tandem mass spectrometer
