# Supplementary figures and images for: In Vivo Efficacy Testing of Peptide Receptor Radionuclide Therapy Radiosensitization Using Olaparib
Source: Cancers (Basel). 2023 Feb 1;15(3):915. doi: 10.3390/cancers15030915 (PMC9913849; doi:10.3390/cancers15030915)

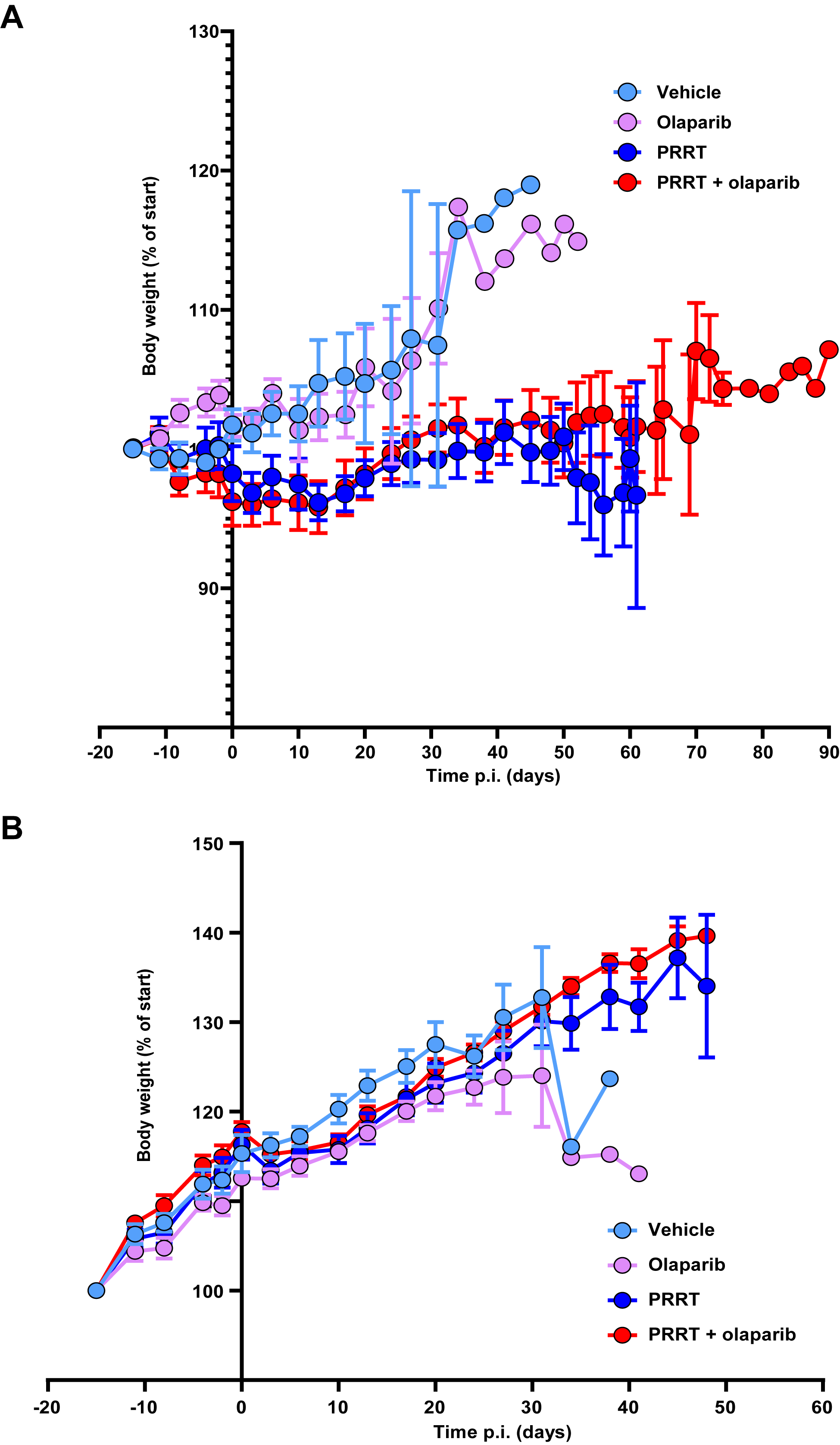

Supplement: Supplementary file 1 [file cancers-15-00915-s001.zip › Supplementary Figure S1.tif]
